# Determinants of Chikungunya and O’nyong-Nyong Virus Specificity for Infection of *Aedes* and *Anopheles* Mosquito Vectors

**DOI:** 10.3390/v15030589

**Published:** 2023-02-21

**Authors:** Solène Cottis, Adrien A. Blisnick, Anna-Bella Failloux, Kenneth D. Vernick

**Affiliations:** 1 Genetics and Genomics of Insect Vectors Unit, Department of Parasites and Insect Vectors, Institut Pasteur, Université de Paris Cité, CNRS UMR2000, F-75015 Paris, France; 2 Graduate School of Life Sciences ED515, Sorbonne Université UPMC Paris VI, 75252 Paris, France; 3 Arboviruses and Insect Vectors Unit, Department of Virology, Institut Pasteur, Université de Paris Cité, F-75015 Paris, France

**Keywords:** chikungunya virus, o’nyong-nyong virus, host–pathogen interactions, vector specificity, *Aedes*, *Anopheles*

## Abstract

Mosquito-borne diseases caused by viruses and parasites are responsible for more than 700 million infections each year. *Anopheles* and *Aedes* are the two major vectors for, respectively, malaria and arboviruses. *Anopheles* mosquitoes are the primary vector of just one known arbovirus, the alphavirus o’nyong-nyong virus (ONNV), which is closely related to the chikungunya virus (CHIKV), vectored by *Aedes* mosquitoes. However, *Anopheles* harbor a complex natural virome of RNA viruses, and a number of pathogenic arboviruses have been isolated from *Anopheles* mosquitoes in nature. CHIKV and ONNV are in the same antigenic group, the Semliki Forest virus complex, are difficult to distinguish via immunodiagnostic assay, and symptomatically cause essentially the same human disease. The major difference between the arboviruses appears to be their differential use of mosquito vectors. The mechanisms governing this vector specificity are poorly understood. Here, we summarize intrinsic and extrinsic factors that could be associated with vector specificity by these viruses. We highlight the complexity and multifactorial aspect of vectorial specificity of the two alphaviruses, and evaluate the level of risk of vector shift by ONNV or CHIKV.

## 1. Introduction

Mosquitoes are considered to be the world’s deadliest animal, indirectly killing millions of people every year by transmitting parasites and viruses. Mosquito-borne diseases caused by either viruses or parasites are responsible for more than 700 million infections each year, and a number of new emerging infectious diseases are caused by arthropod-borne pathogens, most of which are mosquito-borne [1,2]. Although parasites transmitted by mosquitoes continue to be a major scourge on human health, recent decades have witnessed an expansion in the geographic range as well as the public health importance of arthropod-borne viruses (arboviruses). Mosquitoes are globally distributed, except at high latitudes with extreme low temperatures. Nevertheless, climate change has created new possibilities for ecological niche expansion and migration. Thus, new combinations of different mosquito genera and species in the same local regions are increasingly likely, and by sharing the same vertebrate hosts, they also potentially share exposure to similar pathogens including arboviruses. However, even with exposure to arboviruses in host blood, not all mosquito species are competent to be infected and mediate the transmission of all arboviruses, which results from complex interactions between mosquito genetic factors, viral strains, and environmental factors. Consequently, vector specificity underlies vector competence for the transmission of a particular virus or strain. One of the best examples of vector–virus specificity is the transmission of two related alphaviruses, the chikungunya virus (CHIKV) and o’nyong-nyong virus (ONNV), by two distinct mosquito genera, *Aedes* and *Anopheles*, respectively. To date, natural cross transmission, that is, CHIKV by *Anopheles* and ONNV by *Aedes*, has not been reported.

**Figure 1:**
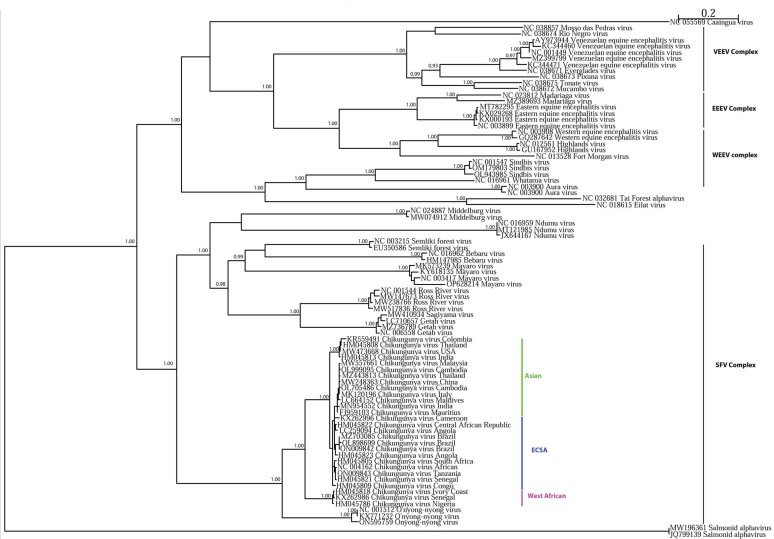
Maximum likelihood tree based on complete genomes of alphaviruses. The alphavirus genomes used here belong to Western equine encephalitis virus (WEEV), Eastern equine encephalitis virus (EEEV), Venezuelan equine encephalitis virus (VEEV), and Semliki Forest virus (SFV) complexes. Among the SFV complex, the various chikungunya (CHIKV) lineages were, respectively, underlined in purple for the West African lineage, in blue for the East/Central/South Africa (ECSA) lineage, and in green for the Asian lineage. The maximum likelihood tree used BioNJ parameters and the GTR model, using Seaview software. Only aLRTs superior to 0,9 are mentioned on the nodes of the tree.

CHIKV and ONNV are phylogenetically related alphaviruses from the family *Togaviridae*, both Old World alphaviruses belonging to the Semliki Forest virus complex (Figure 1). *Aedes* and *Anopheles* mosquitoes belong to two distinct subfamilies of dipterans, *Culicinae* and *Anophelinae*, respectively. *Aedes* mosquitoes are the primary vector of yellow fever virus (YFV), dengue virus (DENV), Zika virus (ZIKV), and other viruses of human health importance, and consequently *Aedes* mosquitoes represent the main vectors of arboviruses [3]. In contrast to *Aedes*, *Anopheles* mosquitoes are the well-known vector for human malaria and, among arboviruses, are thought to be the primary vector of only ONNV [4]. Both mosquito genera are distributed worldwide. Both arboviruses are thought to have originated in Africa, but CHIKV has emerged globally, and caused recent epidemics in the Americas [5], Asia [6], and in Africa [7], while there is no evidence of ONNV transmission outside of Africa [8]. However, there could be biases due to the small number of studies, and the number of ONNV cases in Africa is underestimated due to common symptomatology with CHIKV and the cross-reactivity of diagnostic antibodies [4].

In this review, we gather information about the mechanisms that could drive the vectorial specificity between the vector–virus pairs *Anopheles*/ONNV and *Aedes*/CHIKV. A number of potential factors are summarized in Table 1. We will first describe the vectorial and viral characteristics by focusing on both vectors and viruses. Then, biological and genetic factors of vectors and viruses will be examined, highlighting the vector–virus interactions and the molecular determinants of vector specificity. Finally, we will discuss the role of external factors influencing vector specificity by evaluating potential environmental and human factors that influence CHIKV/ONNV transmission.

### 1.1. Vectorial Systems of Arbovirus Transmission

#### 1.1.1. Sympatric Distribution of *Aedes* and *Anopheles* but Differences in Pathogen Transmission

*Anopheles* mosquitoes are widely distributed in Europe as far north as southern Sweden, Finland, and Russia, as well as at relatively high altitudes such as the French Alps [9,10,11]. In the Americas, *Anopheles* are distributed from northern Canada, throughout the US, Mexico, Central America, and South America, to northern Uruguay. In Africa, a number of *Anopheles* species complexes are widely distributed except in the most arid deserts. Finally, *Anopheles* species complexes are distributed throughout Western Asia, India, Southeast Asia, and the Pacific islands.

*Aedes* mosquitoes are established throughout the Americas (US, Mexico, the Caribbean, and South America) [10]. *Aedes* are distributed throughout much of Africa, except in the most arid deserts. Europe is not highly colonized by *Aedes*, likely because the climate is too wet and cold. However, *Aedes* populations (*Ae. geniculatus*, *Ae. vexans*, *Ae. caspius*, and *Ae. detritus*) have been established for decades in the Mediterranean regions of Italy, Spain, southern France, Greece, and Croatia [3], and these may be potential vectors for emergent viruses [12]. In Asia, southern regions (India, Bangladesh, and Southeast Asia) are favorable to *Ae. albopictus* and *Ae. aegypti* establishment. Some *Aedes* species (*Ae. Geniculatus*) are also found in central Asia and Russia. Other *Aedes* species (*Ae. niveus and Ae. japonicus*) are found in China and Japan, and Southeast Asia. One *Aedes* species (*Ae. vexans*) is found in almost all Northern Hemisphere countries, Southeast Asia, and Australia. Thus, much of the world displays sympatric distribution of these two mosquito genera.

Many mosquito arboviruses have an African origin, including CHIKV, ONNV, Zika virus (ZIKV), DENV, and yellow fever virus (YFV) [10]. *Aedes aegypti* and the *Anopheles gambiae* species complex (including the major taxa *An. gambiae* sensu stricto and *Anopheles coluzzii*, hereafter generally named collectively as *An. gambiae*) are the main anthropophilic species responsible for the transmission of human pathogens in Africa. While *Ae. aegypti* and *An. gambiae* mosquitoes transmit shared pathogens (e.g., *Wuchereria bancrofti* [13,14]), they clearly differ in the transmission of arboviruses. While *Ae. aegypti* transmit arboviruses from multiple viral families such as *Flaviviridae* (ZIKV, DENV, YFV, and JEV), or *Togaviridae* (CHIKV and Mayaro virus), *An. gambiae* mosquitoes mainly transmit parasites belonging to *Plasmodium* spp. and are the primary vector of just one arbovirus, the alphavirus ONNV (*Togaviridae*). Naturally present in Africa, these mosquito species share a large geographical distribution and, necessarily, the same human hosts, but *Aedes* appear to be more physiologically competent to transmit viruses.

Both *Ae. aegypti* and *An. gambiae* are highly anthropophilic, and both are exposed to arboviruses in the blood of infected human hosts, so behavior does not explain the difference in their viral transmission profiles. Thus, the explanation for vector specificity of viral transmission most likely lies in differences in vector immunity or other host cellular and physiological factors, virus genetics, or other factors. Most arboviruses have an RNA genome, and RNA polymerases lack proofreading activity leading to high mutation rates during replication [15]. This generates a swarm of viral particles that are genetically different but share the same consensus sequence, called quasispecies. Viral diversity contributes directly to viral evolution, as exemplified by a CHIKV genotype carrying a single point mutation in the E1 protein that enabled efficient replication and transmission in a novel vector, which enhanced the vector competence of *Ae. albopictus* for CHIKV [16]. This example illustrates that the mosquito vector is a selective filter acting on quasispecies, making the vector not a simple flying syringe but rather an important driver of viral genome evolution.

#### 1.1.2. O’nyong-Nyong and Chikungunya, Two Closely Related Alphaviruses

Alphaviruses belong to the *Togaviridae* family and are enveloped viruses with a positive single-strand non-segmented RNA genome of 10–12 kb. The known arbovirus members of the *Togaviridae* are grouped within the genus *Alphavirus,* which are present on all continents except Antarctica [17]. CHIKV and ONNV belong to the Semliki Forest virus complex, which is comprised of Semliki Forest virus (SFV), CHIKV, ONNV, Ross River virus (RRV), Mayaro virus (MAYV), Sindbis virus (SINV), and bebaru virus (BEBV). In this complex, ONNV and CHIKV are the closest members genetically [18].

In the Tanzanian Makonde language, chikungunya means “disease that bends up the joints” [19]. CHIKV was first reported in 1952 in Tanzania, and the first CHIKV strain was isolated in 1953 [20,21]. The virus was detected in sylvatic mosquitoes such as *Ae. africanus*, *Ae. furcifer*, and *Ae. taylori*, as well as in non-human primates both in Uganda and Tanzania, pointing to an origin in Central and East Africa [19]. Since its recognition in 1952, CHIKV has caused many outbreaks in Africa and Asia, and in the last 20 years has spread globally as an epidemic threat [19,22]. A recent CHIKV outbreak in the Republic of Congo in 2019 was caused by novel strains harboring new mutations in the envelope surface proteins E2: E2-T126M and E2-H351N [7].

The name ONNV comes from the language of northwestern Uganda meaning “very painful weakening of joints disease”. The first ONNV strain was isolated in 1959 from the serum of febrile patients in Gulu, Uganda, during the first-known outbreak of 1959–1962, which included Uganda, Kenya, Tanzania, Malawi, Mozambique, Democratic Republic of Congo, Central African Republic, Cameroon, and Senegal, with more than 2 million diagnosed cases [4,8,23]. ONNV was first identified in 1952. In addition, because the human disease is essentially the same and the viruses cross-react using most serological diagnostics, there is likely a high rate of misdiagnosis [8].

**Figure 2:**
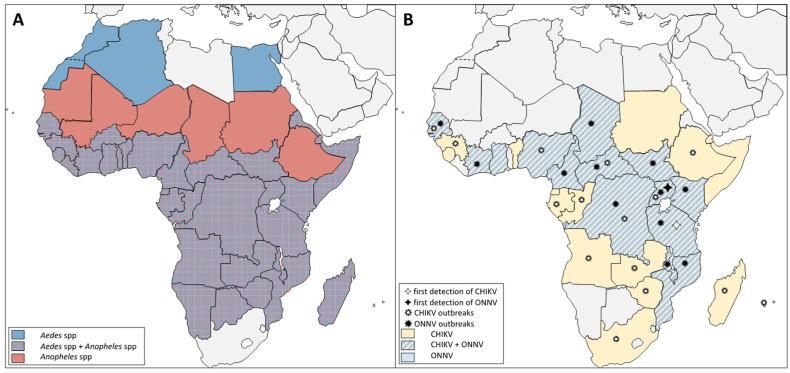
African distribution of *Anopheles* and *Aedes* vectors and the alphaviruses ONNV and CHIKV. (**A**) *Aedes* mosquitoes were established in North Africa but are absent from other regions with *Anopheles* populations. *Aedes* and *Anopheles* vectors are generally sympatric in the Sub-Saharan region. (**B**) ONNV and CHIKV have been reported in most of Sub-Saharan Africa. ONNV reports are restricted to countries where CHIKV is also present, and countries such as South Africa and Chad have detected CHIKV cases, but *Aedes* vectors have not been detected in these regions. References of ONNV’s and CHIKV’s first detection and outbreak cases are cited in the text.

There is sympatry of both *Aedes* and *Anopheles* anthropophilic mosquitoes as well as ONNV and CHIKV viruses throughout much of the African continent, including Senegal [23,24], Ghana [29], Chad [30], Central African Republic [31], South and North Sudan, Mozambique [32], Malawi, Ivory Coast [23], Nigeria [23,24], Cameroon [23,24], Democratic Republic of Congo [7,23,33], Kenya [23,24], and Tanzania [23,24]) (Figure 2). The geographical distribution of ONNV, putatively limited to Africa, and the worldwide distribution of anthropophilic *Anopheles*, including former human malaria vectors in zones where malaria parasites were eliminated by medical intervention, provoke the question about the factors that could explain the geographical restriction of *Anopheles* arbovirus transmission to a single virus in one continent.

### 1.2. Mosquito Intrinsic Factors and Virus Transmission

#### 1.2.1. *Aedes* and *Anopheles* Genetics and Evolution

The evolutionary divergence between *Aedes* and *Anopheles* took place approximately 145 million years ago (mya) [34,35]. The last common ancestor of Old World (including the highly anthropophilic *An. gambiae* complex of African malaria vectors) and New World Anopheles was about 100 mya [36]. The origin of the current anthropophilic *Ae. aegypti* occurred in Africa about 10,000 years ago as a result of an unknown selective event allowing the adaptation of a forest-dwelling and probably zoophilic ancestral form to the human peridomestic niche [37,38]. The descendants of this selection event and population bottleneck then spread throughout the world only several hundreds of years ago to become the cosmopolitan *Ae. aegypti* vector of today. Thus, the most anthropophilic and efficient human disease vectors in the world, *Ae. aegypti* and the *An. gambiae* complex, both arose in Africa, and have evolved together there since then.

The two mosquito genera display different genome sizes (Table 1). The genome of *Ae. aegypti* is 1380 megabases (MB) [30], while that of *Anopheles* is 278 MB [39]. This genome size difference is partly explained by the different density of transposable elements (TEs), which occupy 50% and 16% of the genomes of *Ae. aegypti* and *An. gambiae*, respectively [39,40]. Consequently, *Aedes* chromosomes are 2.3 times longer than in *Anopheles* [41].

These two mosquito taxa share 67% of orthologous proteins, with an average peptide identity encoded by single-copy orthologous genes of 74% [30]. More precisely, the two mosquitoes share approximately 2000 gene orthologs which can be said to represent the central set of genes governing mosquito biology, although, of those, only 250 have a known function [30]. *Ae. aegypti* displays enrichment as compared to *An. gambiae* in genes encoding zinc finger proteins, insect cuticle, cytochrome P450, odorant binding proteins, insect allergen-related proteins and high mobility group domains (HMGB-I and HMGB-Y) [30]. HMGB-I-domain-containing proteins were reported to be associated with the formation of a ternary complex of DNA, Rel1, and NF-kB [42]. In this complex, HMGB-I acts as the potentiator of Rel1 DNA-binding and transcriptional activation by bending DNA at the binding site of the complex, and could be related to Toll pathway activity and antiviral immunity. Regarding zinc-finger-containing proteins, the Veneno Tudor protein of *Ae. aegypti* promotes the expression of a class of small RNAs in the RNA interference (RNAi) pathway, the P-element-induced wimpy testis (PIWI)-interacting RNAs (piRNAs), known to be important for anti-CHIKV or anti-ONNV pathways in their respective vectors [43,44,45].

#### 1.2.2. ONNV and CHIKV Genetics and Evolution

The symptoms of the human diseases caused by ONNV and CHIKV are indistinguishable, which is consistent with the likelihood of a common ancestral origin followed by subsequent divergence. The hypothetical viral ancestor was probably transmitted among vertebrates such as non-human primates by *Aedes* mosquitoes [46,47,48]. ONNV was associated with periodic outbreaks in Africa in which *Anopheles* mosquitoes were implicated as vectors, suggesting a potential history of adaptation of a common ancestor first to *Aedes* and then to *Anopheles*, followed by the independent evolution of the two viruses [46].

The high viral mutation rate produces a cloud of quasispecies as mentioned previously. Viral variants can expand if they express new adaptive phenotypes for traits such as virulence, pathogenicity, and/or immunogenicity [49,50]. Moreover, viral variants may also increase in frequency without selection for advantageous alleles (e.g., genetic drift). For arboviruses, quasispecies production during the transmission cycle allows for more efficient adaptation to their two very different hosts, vertebrates, and mosquitoes [51]. ONNV quasispecies display an equilibrium of both an arginine codon (CGA) or a stop codon (UGA) as nucleotide variants between the nsP3 and nsP4 genes [52,53,54]. The presence of the arginine codon confers higher viral production in mammalian and *Ae. albopictus* cells, while the stop codon confers higher fitness for viral infectivity in *An. gambiae* [52,53]. Unlike in ONNV, most CHIKV strains harbor the stop codon at the homologous position, although some viral isolates carry the Arg codon, but the polymorphism does not influence CHIKV replication in either mammalian or insect cells [55,56]. Thus, the Arg-to-stop codon mutation has more influence on ONNV replication as compared to CHIKV, and particularly in the *Anopheles* host.

### 1.3. Vector–Virus Interactions

#### 1.3.1. The Virus Cycle in the Mosquito Vector

Multiple essential steps are required for a successful viral cycle in an infected mosquito resulting in infectious viral particles that can be transmitted to a vertebrate host during a bloodmeal.

##### Primary Midgut Infection

Blood from a virus-infected vertebrate host first enters the mosquito midgut lumen during a bloodmeal. After cell attachment, the successful virus enters epithelial cells via receptor-mediated endocytosis [57]. In *Ae. aegypti*, two glycosylated proteins of 38 and 60 kDa in the membranes of brush border cells were identified as susceptibility factors to CHIKV infection, as they are in lower concentrations in refractory mosquito populations than in susceptible ones [58]. In addition, some other putative receptors of 24, 45, 58, 60, and 62-kDa were also identified in the membrane fractions of the *Ae. albopictus* cells, with potential orthologs in *Ae. aegyti* [58]. Initially found at the mitochondrion surface, the ATP synthase was also identified as a cell membrane protein in many cell types, including in *Ae. aegypti* cells [59]. In studies to identify candidate receptors for DENV-2 in the *Aedes* midgut, ATP synthase β(ATPSβ) was suggested to function as an ATP provider facilitating the function of the HSc 70 chaperon, which could help to accumulate virus particles on the membrane [60]. The analysis of the role of ATPSβ in the CHIKV infection of *Ae. Aegypti* cells also revealed the critical role of ATPSβ since inhibition with antibodies decreased up to 30% of the fraction of CHIKV-infected cells. These results were supported by the impact of the ATPSβ synthase on the number of infected cells. The colocalization of ATPSβ and the CHIKV E2 protein also strongly corroborates the likely role of ATPSβ during viral infection [59].

Similarly, a quantitative proteomic study of ONNV-infected *An. gambiae* detected elevated protein abundance of a 230 kDa cadherin and an ortholog of Rab5 in infected mosquitoes [61]. Cadherins on the *Aedes* cell surface bind to West Nile virus (WNV) and DENV envelope proteins, which suggests the involvement of mosquito cell cadherins as entry factors during arbovirus infections [61,62,63]. Moreover, Rab5 integrity is also important for the CHIKV infection of mammalian cells [63].

##### Midgut Escape Barrier

The mosquito midgut is comprised of a single layer of epithelial cells surrounded by a basal lamina comprised of laminin, collagen IV, and other proteins and glycans [64]. The basal lamina is highly permeable but may physically limit the number of viral particles able to disseminate into the mosquito body [65]. CHIKV escape from the midgut epithelium occurs before 48 h post-bloodmeal [57,64,66,67]. An electron microscopic study detected that CHIKV particles accumulated in the midgut between 24 and 32 h post-bloodmeal [64]. ONNV was still restricted to the *Anopheles* midgut at 3 days post-bloodmeal but was detected in salivary glands, legs, and circulating cells perfused with hemolymph at 7 days post-bloodmeal, indicating infectivity by this time point [68]. Thus, ONNV in *Anopheles* has a longer extrinsic incubation period, as it has not yet disseminated into the hemocoel before 3 days post-bloodmeal, while CHIKV is present in the salivary glands by 2 days post-bloodmeal.

##### Viral Dissemination and Transmission

Following escape from the primary infection in the midgut epithelium, both viruses disseminate to all of the tissues exposed to the hemocoel, such as the fat body, hemocytes, and salivary glands, and can also initiate a secondary infection of the midgut epithelium. To infect the salivary glands, virus particles diffuse across the basal lamina of the glands and infect acinar cells, where viruses replicate and are then released into the apical cavities, where they remain until being released into the salivary duct during the bloodmeal [65,69,70]. Once CHIKV and ONNV viruses infect the salivary glands, viral transmission is possible during blood feeding. The salivary glands are the second critical bottleneck, after the midgut, essential for vector competence, and represent the second filter and potential barrier for the virus [51,57,65].

A quantitative proteomic analysis of CHIKV-infected *Ae. aegypti* and ONNV-infected *An. gambiae* midguts 6–7 days post-bloodmeal detected the modulation of 32 or 22 proteins, respectively, involved in multiple metabolic pathways [61,71]. Even though analyzing midgut tissues, these time points represented the period of the disseminated infection in both mosquitoes, and not the primary midgut infection.

The longer incubation period before midgut escape and dissemination for ONNV in *Anopheles* as compared to CHIKV in *Aedes* might be explained if there are differentially efficient antiviral mechanisms that could retard the production of viral particles and therefore the speed at which viruses can overcome physiological and physical barriers in *Anopheles* as compared to *Aedes*. The importance of these barriers was investigated in a comparison of *An. gambiae* and *Ae. aegypti* vector competence towards three different strains of ONNV and one strain of CHIKV [72]. Some strains of ONNV were able to infect, disseminate, and reach *Aedes* mosquito saliva. Conversely, when infecting *Anopheles* with CHIKV, at Day 7 post-bloodmeal, very few mosquitoes harbored virus particles in the hemocoel, with low viral titers, and no mosquitoes had CHIKV-positive saliva [72].

#### 1.3.2. Mosquito Immunity and Antiviral Mechanisms

##### Mosquito Innate Immunity

The vertebrate bloodmeal hosts of mosquitoes can harbor various microorganisms, including bacteria, parasites, and viruses. Insects, including mosquitoes, have evolved four main immune signaling pathways to control pathogen infections, whether environmental or blood-borne: Toll, IMD, JAK/STAT, and RNA-interference (RNAi) pathways. These pathways are activated by the stimulation of upstream ligands and receptors by different pathogen elicitors and infection signals. However, some of the upstream ligands and most of the elicitors that they recognize are indirect and mostly still unknown. This is distinct from the mechanism of vertebrate Toll-like receptors (TLRs), in which cell-surface TLRs bind specifically to the pathogen elicitors that they recognize and directly transduce a cellular signal upon elicitor stimulation.

The Toll pathway can control viral infection in mosquitoes, and is also activated by viral infection as observed for ZIKV, CHIKV, and DENV in *Aedes* [25,73,74] and for ONNV in *Anopheles* [68,75]. The IMD pathway can be activated by cell binding by viruses [76] but also by the mosquito intestinal microbiota [26,68,73]. In *Aedes* mosquitoes, CHIKV infection is not limited by the Toll pathway; however, Toll activation is inhibited by CHIKV [73]. Toll limits ONNV infection and ONNV inhibits Toll activation in *Anopheles* hemocyte cells [68]. Finally, the RNAi pathway responds to viral infection through the generation of small interfering RNA (siRNAs) targeting short sequences of viral genomes. RNAi controls both viral replication and dissemination as reported for alphaviruses such as ONNV in *Anopheles* [44,68] and CHIKV in *Aedes* [73], or flaviviruses such as ZIKV and DENV [27,77].

The first line of defense encountered by a pathogen in mosquitoes and other invertebrates is often the soluble immune factors secreted by invertebrate immune cells, the hemocytes, which are cells that circulate or attach to surfaces in the hemocoel. The expression of these immune factors is differentially controlled by the Toll, IMD, and JAK/STAT pathways, and the factors serve as sentinels and activators of more complex pathways to maintain organismal homeostasis. Soluble leucine rich repeat (LRR) proteins such as LRR immune proteins (LRIMs), as well as thioester-containing proteins (TEPs) in the hemolymph, can form protein complexes associated with malaria parasite neutralization in *Anopheles* [28,78,79,80,81,82]. To date, no such complexes have been described in *Aedes*, despite similar numbers of TEPs in both mosquito genera (respectively, 24 in *Anopheles* and 29 in *Aedes*) [83]. However, due to the importance of the *Anopheles* LRIM1/APL1C complex in immunity to malaria parasites, these molecules were also examined for a role in antiviral immunity. In *Anopheles*, the LRR molecules APL1A and APL1C are protective against the ONNV primary midgut infection, and orthologs in *Aedes* were transcriptionally regulated at 24–72 h post-CHIKV infection, suggesting a potential antiviral role that has not been further studied [84].

Mosquito antiviral immunity is physiologically compartmentalized in *Anopheles* infected with ONNV [64]. In the primary midgut infection 3 days post-bloodmeal before midgut escape, the IMD and JAK/STAT pathways are strongly antiviral, in part due to an effect of the enteric microbiome, but the RNAi pathway plays no antiviral role. In contrast, after the establishment of the disseminated systemic infection, the Toll and RNAi pathways are strongly protective. To our knowledge, the compartmentalization of antiviral immunity has not yet been examined in *Aedes* mosquitoes.

##### Viral Tolerance

Infected hosts can also respond to pathogens by mechanisms that produce tolerance, rather than resistance [85]. Resistance mechanisms reduce the pathogen load, at most leading to elimination and sterile immunity, while tolerance mechanisms reduce the fitness cost of infection without influencing the pathogen load. In mosquitoes, tolerance towards viral infection and particularly to high viral load may be linked to the generation of viral-derived DNAs (vDNA). The vDNAs are produced following a viral infection as found in *Ae. aegypti* challenged with CHIKV [86,87]. Viral tolerance in mosquitoes based on vDNA may be associated with the piRNA pathway [88]. Genomic studies of dipteran genomes revealed the presence of seven PIWI proteins in *Ae. aegypti*, and only one in *An. gambiae*, which may indicate a greater diversity in PIWI pathway proteins in *Aedes* than in *Anopheles* [89]. In addition, non-retroviral integrated RNA virus sequences (NIRVs) are found in clusters of PIWI RNAs in the vector genome, highlighting a potential link of PIWI RNAs with NIRVs [90,91]. NIRVs in mammalian cells can generate translated proteins that interfere with the replication of related viruses [92], and could potentially play a similar role in mosquitoes. In *Ae. aegypti*, 50% of NIRVs are integrated close to PIWI RNA clusters. There may be fewer genomic NIRVs in *Anopheles* than in *Aedes* [90].

#### 1.3.3. Viral Factors Underlying Host Specificity

To initiate infection, virus particles must first attach to the host cell surface by an interaction of the viral envelope protein with extracellular host proteins or other factors. ONNV was able to infect both *Ae. albopictus* and *An. gambiae* cell lines but not an *Ae. aegypti* line [72,93]. CHIKV was replicated only in *Ae. aegypti* and *Ae. albopictus* [94] but not in *An. gambiae* [72]. Both CHIKV and ONNV display a broad cellular tropism, as they can infect a large range of cell types in vertebrates and invertebrates [68,95,96]; however, differences in host cellular receptors may be a part of the explanation for vector species specificity for *Aedes* or *Anopheles.* Using ONNV/CHIKV chimeric constructions, it was found that only chimeras with ONNV structural proteins are able to infect *Anopheles* cells [97]. Therefore, all of the viral structural proteins of ONNV appear to play a role in its infection specificity for *Anopheles* cells.

The use of multi-plasmid combinations allowing the replication and transcription of the viral RNA by the non-structural proteins (termed a trans-replicase system) indicated that the non-structural proteins of ONNV were not able to support the replication of ONNV RNA in *Ae. albopictus* cells [98]. However, the replication and transcription of ONNV RNA by CHIKV and MAYV non-structural proteins was observed in those cells. Taken together, these results suggest that differences in cellular factors required for replication and/or cellular antiviral mechanisms are one of the determinants of specificity.

The viral non-structural protein 3 (nsP3) is known to be a critical factor both for CHIKV and ONNV cellular infection, and could at least partly underlie vector specificity [99]. Replacing the CHIKV nsP3 gene with ONNV nsP3 in the CHIKV genomic backbone allowed up to 63% infectivity for *Anopheles* mosquitoes, while the CHIKV backbone carrying its own nsP3 gene is noninfective to *Anopheles* [99]. This result suggests that ONNV nsP3 is required specifically for *Anopheles* infection. Thus, the differences in ONNV and CHIKV nsP3 sequences and host cellular protein partners could function as host restriction factors. nsP3 is comprised of three domains, and in order from N to C-terminal they are as follows: the macro-domain (MD) possessing a phosphatase and RNA-binding activity [100], the alphavirus unique domain (AUD) harboring a zinc-binding function [101], and the hypervariable domain (HVD) [54]. The main divergence between the nsP3 of CHIKV and ONNV resides in the HVD. This domain is highly phosphorylated and intrinsically disordered [102], lacking a defined secondary structure [103]. The presence of only the HVD of ONNV nsP3 swapped into the CHIV backbone allows for minimal infection of the chimeric virus in *An. gambiae* [99].

In both insect and mammalian cells, CHIKV’s and ONNV’s nsP3 interact by means of their FGDF domain with the NTF2-like domain of the host cell factor, Ras GAP SH3-domain-binding protein (G3BP, or the mosquito ortholog Rasputin) [54,104,105,106,107]. G3BPs are RNA-binding proteins involved in stress granule formation, but their cellular functions are not well understood [54,104,105,106,107]. The interaction between nsP3 and GFBP/Rasputin is conserved among alphaviruses both in mammalian and insect cells [106,107,108,109]. Rasputin in *Ae. albopictus* plays a proviral role for CHIKV infection [107]. Despite overall conservation, Rasputin proteins of *An. gambiae* [AGAP000403] and *Ae. aegypti* [AAEL005528] display only 66% peptide identity. ONNV’s and CHIKV’s nsp3 colocalize with Rasputin when expressed in lepidopteran Sf21 cells [107] in the *Ae. albopictus* C6/36 cell line [108] and in the *Ae. aegypti* Aag2 cell line [104]. However, these interactions have not yet been investigated in *Anopheles*, and the interaction between the Rasputin of *Anopheles* with the nsP3 from ONNV or CHIKV using an in vitro approach could provide an insight into the role of Rasputin-nsP3 in the vectorial specificity of the viruses.

Other partners of CHIKV nsP3 in *Ae. aegypti* identified by co-immunoprecipitation include RM62F, a DEAD-box containing factor of the Ago-RISC complex [44,110]. In mammalian cells, viral nsP3 interacts with many proteins [54]; thus, it is not excluded that orthologs of these partners in mosquitoes could also interact with nsP3 in vectors as observed for G3BPs.

### 1.4. Environmental and Human Host Factors

#### 1.4.1. Biotic and Abiotic Factors Influencing Viral Transmission

##### Biotic Factors

Mosquitoes harbor an enteric microbiome composed of bacteria, fungi, and viruses that can influence their biology, including vectorial capacity. The composition and density of the microbial consortium depends on the vector species and its environment, and can differ between individuals within a population.

##### Co-Infection with Other Pathogens

The presence of other microbes in the vector, in particular viruses, could modify susceptibility to infection and the capacity for the transmission of ONNV or CHIKV. Regarding *Ae. aegypti*, CHIKV could co-infect with other arboviruses known to be sympatric and co-circulating, such as YFV [111], ZIKV [112,113], or JEV [114]. The co-circulation of these flaviviruses with CHIKV in the same geographical area [115] has been highlighted by the co-infection of patients with ZIKV and CHIKV [116]. CHIKV and DENV co-infected mosquitoes have been collected during outbreaks [113,117], including close to the houses of co-infected patients [118]. Mosquitoes co-infected with ZIKV and CHIKV can simultaneously transmit both viruses by a single bite [119]. Regarding *Anopheles*, mosquitoes in nature could be co-infected with ONNV and/or the *Plasmodium* parasite in areas of co-circulation. *An. gambiae* co-infected with ONNV and *Plasmodium berghei* displayed reduced numbers of melanized malaria parasites [75], while *Anopheles* co-infected with *Trypanosoma* and *Plasmodium* displayed elevated numbers of malaria parasites [120]. Neither of these pathogens were tested for an effect on ONNV, but clearly the outcomes of co-infection are case-dependent and need to be determined empirically. The influence of *Anopheles* co-infection with other pathogens upon either ONNV or CHIKV infection requires investigation. A longitudinal survey of the human population in Kenya detected rates of seropositivity to ONNV above 20% during an inter-epidemic period [8]; however, a survey of febrile children in Kenya detected Plasmodium but not ONNV infection, an apparent inconsistency with the longitudinal survey that could not be explained [121]. In a laboratory study, the co-infection of mice with *Plasmodium* caused reduced ONNV viral load and associated viral pathologies, probably due to a protective effect of *Plasmodium*-induced interferon gamma [122].

##### Superinfection Exclusion

Due to the sympatric geographical distribution of ONNV and CHIKV, co-infections of patients are not rare; in a survey in Kenya, 38% of people seropositive for ONNV or CHIKV displayed high titers for both alphaviruses [8]. In superinfection exclusion (also called homologous interference), a second viral infection is inhibited in cells previously infected by the same virus or a closely related virus. CHIKV superinfection exclusion was seen in mammalian cells with CHIKV, Sindbis virus (SINV), and even with influenza A virus [123]. Superinfection exclusion was also observed for CHIKV in MAYV-infected *Ae. aegypti* mosquitoes but interestingly not for MAYV in CHIKV-infected mosquitoes [124]. Superinfection exclusion by or towards CHIKV and ONNV can occur with other arboviruses [123,124]. Therefore, superinfection exclusion could potentially influence vector specificity between ONNV and CHIKV.

##### Mosquito Virome

Recently, more attention has been paid to insect-specific viruses (ISVs) for their potential role in mosquito biology and arbovirus transmission. ISVs belong to taxonomically diverse viral families and include viruses with DNA or RNA genomes [125]. *Ae. aegypti* has a diverse and abundant virome [126,127,128], as does *Anopheles* [125,129,130], with at least 51 viruses found in *Anopheles*, including arboviruses, reported to replicate in vertebrate cells [125]. Although *Anopheles* mosquitoes are believed to be less efficient vectors of viruses, the presence of a rich RNA virome indicates the absence of an intrinsic blockade in *Anopheles* to RNA virus infection and propagation [131,132]. Thus, because both *Aedes* and *Anopheles* mosquitoes maintain complex viromes of RNA viruses, the ISV virome appears unlikely to underlie or explain their contrasting vector competencies for arboviruses.

The evidence that ISVs can mediate superinfection exclusion for arboviruses, or for other ISVs, was first highlighted by studying the effect of the ISV Culex flavivirus (CxFV) on WNV in *Culex* mosquitoes [133]. Superinfection exclusion was observed in *Ae. aegypti* both in vitro and in vivo between Eilat virus, a mosquito-specific alphavirus, and CHIKV [134]. Phasi charoen-like virus (PCLV) infection of *Ae. albopictus* cells inhibited ZIKV, DENV, and La Crosse viruses [135]. The infection of *Ae. aegypti* mosquitoes with the ubiquitous insect-specific flavivirus, cell-fusing agent virus (CFAV), reduced the dissemination in vivo of DENV and ZIKV [136]. The unclassified ISV, Negev virus, was able to induce superinfection exclusion with CHIKV or ONNV in *Ae. albopictus* cell culture [137]. Finally, there can be dynamic interactions between ISVs. In *Anopheles*, the abundance in vivo of two ISVs, the dicistrovirus Anopheles C virus (AnCV) and Anopheles cypovirus (AnCPV), was inversely correlated in individual mosquitoes, and the two ISVs were differentially affected by the Toll and JAK/STAT immune pathways [132,138]. Therefore, ISVs represent a level of complexity in arbovirus vector competence and immunity that is still poorly understood, and more detailed studies are needed to investigate whether ISVs specific to either *Aedes* or *Anopheles* could influence vector specificity.

##### Mosquito Bacterial Microbiota

The enteric bacterial flora, or microbiome, is another mostly non-genetic factor that is important for mosquito biology, and can influence viral infection as the midgut is the first mosquito barrier to blood-borne pathogens [68,139]. The enteric microbiome is diverse and complex, and the consortium of bacterial taxa in the midgut is dependent on environmental, physiological, and biological factors, including the effect of a bloodmeal. Geography and ecological settings strongly affect microbiome composition [140,141,142,143,144]. However, different mosquito species collected from the same site can display differences in their bacterial flora, and the same species from different sites can also be very different [143,145]. Colonies of *Ae. Aegypti* and *An. Gambiae* exposed to the same conditions in the laboratory displayed different microbial profiles [142], but this result does not necessarily indicate species-specific microbiota, because different colonies of *An. Coluzzii* raised in the same facility also displayed differences in their bacterial microbiomes, likely indicating genetic differences that influence the preferential carriage of specific bacterial taxa [143]. In addition to the bacterial microbiome, mosquitoes also harbor a microbiome of eukaryotic microbes and this has barely been examined. A field study of African *Anopheles* revealed a diverse and variable eukaryotic microbiome, including taxa related to pathogens such as *Plasmodium*, which could have the potential to influence host immunity and possibly superinfection exclusion [143].

ONNV infection of *Anopheles* requires the presence of live enteric flora, and is antagonized by the antibiotic treatment of mosquitoes [68]. This result is surprising because *Plasmodium* infection of *Anopheles* is inhibited by the enteric bacterial flora, and augmented by antibiotic treatment [146,147,148]. It was shown that a key *Anopheles* LRR immune factor, APL1, exerts a strong influence on the composition of the bacterial flora, which indicates a link between immunity and microbiome [149]. CHIKV infection of *Aedes* can also be influenced by the midgut fauna, because co-infection with the parasitic worm *Dirofilaria immitis* enhances CHIKV infection, while the presence of *Wolbachia* inhibits CHIKV infection and dissemination [69,150].

#### 1.4.2. Abiotic Factors

Abiotic factors are the non-living components of an ecosystem. These external factors are primarily physical factors related to the environment such as temperature, light, water quality, and rainfall, that may influence mosquito biology and the ability to transmit viruses.

##### Temperature and Weather

Mosquitoes are poikilothermic organisms, with an internal body temperature that essentially mirrors the ambient environmental temperature. Consequently, temperature and weather directly influence mosquito metabolism and connected life traits. The optimal temperature window that maximizes organismal fitness varies according to the ecological niche of the organism, for example, temperate or tropical, so it is difficult to generalize [151,152,153]. Temperature also directly controls the rate of virus replication in the mosquito vector, and host processes such as immunity as well, with an optimum range for transmission that integrates many factors. The conditions for transmission of CHIKV by *Aedes* peak between 26 °C and 29 °C [154,155,156]. Interestingly, temperature optima can diverge for different viruses in the same mosquito, because *Ae. albopictus* is competent to transmit CHIKV at 20 °C and 28 °C, but the species is only competent to transmit DENV at 28 °C but not 20 °C [157]. The role of temperature on ONNV infection and transmission in *Anopheles* has not been investigated.

In addition to temperature, *An. gambiae* and *Aedes* mosquito longevity and survival are positively correlated with rainfall and humidity [158,159,160], and a positive correlation was observed between rainfall and CHIKV incidence in India [161]. Finally, the aquatic larval stages of mosquitoes are exposed to physicochemical parameters including pH, salinity, and others [10,162]. Both *Anopheles* and *Aedes* mosquitoes lay their eggs in different kinds of larval sites, with undoubtable differences in chemical composition and effects on mosquitoes.

### 1.5. Role of Human Hosts

#### Host Population Genetics

Human host factors are unlikely to be relevant for the vector specificity of CHIKV and ONNV, especially since the pathologies are almost identical, and mixed human infections are frequent. Thus, there is no evident reason to postulate a selective pressure in humans that could influence vector specificity. It can be noted though that Africa hosts one of the two largest rainforests in the world, the Congo Basin, which is a hot spot for biodiversity and potential zoonoses [163]. Exposure to multiple viral pathogens may influence the outcome of new viral infections. Type I interferon (IFN) is released after viral infection [164]. IFN allows the formation of the cytosolic complex, ISGF-3, which promotes IFN-stimulated genes (ISGs) involved in many cellular processes such as RNA processing, protein stability, and cell viability, and also affects, in particular, virus replication. Among the ISGs are the interferon-inducible transmembrane (IFITM) gene family, implicated in antiviral responses to numerous viruses including influenza HIV, RSV, DENV, and alphaviruses including CHIKV and ONNV [165]. IFITM3 can inhibit alphavirus infections at primary stages by inhibiting virion fusion with the cell surface and pH-dependent membrane fusion required for endocytosis, and can restrict viral proliferation at secondary stages by modulating viral particle production through the limitation of both pro-inflammatory cytokine and chemokine secretion as well as the number of CHIKV antigen positive macrophages and neutrophils [165]. Due to the importance of IFITM3 in viral infection, its polymorphism is critical for an adapted response of the innate immune system to viral infections. The minor allele (A) of IFITM3, rs34481144 SNP, is genetically associated with severe Influenza A infection and is present in European populations at a frequency of 45% [166].

## 2. Conclusions

In Africa, the two closely related alphaviruses CHIKV and ONNV, as well as their respective vectors, *Aedes* and *Anopheles* mosquitoes, are generally sympatric and share highly anthropophilic feeding behavior. Laboratory studies indicate that there is specificity of viral transmission by the vectors for CHIKV and ONNV, but detailed field studies that could rule out cross-transmission are lacking. Based on experimental studies, the vector specificity for ONNV or CHIKV probably results from a combination of factors presented here, particularly interactions between Rasputin and nsP3. Even though exceptional, vector shifts by a pathogen may occur, as exemplified on La Reunion Island where a single mutation in the CHIKV genome enhanced viral transmission by a new vector, *Ae. albopictus*. The risk of the emergence of ONNV outside of Africa, or of augmented arbovirus transmission by *Anopheles*, could become possible as climate change modifies mosquito distribution, physiology, lifespan, and potential exposure to new pathogen profiles.

## Figures and Tables

**Table 1:** Summary of potential factors underlying CHIKV and ONNV vector specificity.

**A. Viral comparisons**			
		**ONNV**	**CHIKV**
**Viral classification**	Family	*Togaviridae*	
	Genus	*Alphavirus*	
	Antigenic complex group	Semliki forest complex	
**Viral genome**	Group	Baltimore group IV	
	Type	ssRNA (+)	
	Nucleic sequences	76.48% identity with 93% of coverage	
	Mutation rate	CHIKV: average estimation of 4.33 × 10^−4^ nucleotide substitutions per site per year [9] ONNV: no estimation	
**Viral cycle in cells**		similar; same viral proteins harbor similar functions	
**Geographic distribution**		Sub-Saharan region (Africa)	
**Protein sequence**	Opal-to-Arg codon between nsP3 and nsP4	Equilibrium of Opal-to-Arg codon [10,11]	Minority of Arg codon [12,13]
**Geographic distribution**		Restricted to Africa	Found in four continents (America, Africa, Europe and Asia
**Mortality rate in patients**		not reported	0,1% of cases [14]
**Vectors used for transmission**		*Anopheles funestus* *Anopheles gambiae* *Aedes aegypti* [15] *Mansonia uniformis* [11]	*Aedes aegypt* *Aedes albopictus*
**B. Mosquito vector comparisons**			
		***Anopheles gambiae***	***Aedes aegypti***
**Behavioral traits**	Blood feeding preferences	Anthropophilic	
	Blood feeding time	Crepuscular or nocturnal	
	Blood feeding places	Endophilic	
**Developmental stages**	Laying sites	Clear, unpolluted, fresh or salt water	Walls of water containers
	Larval habitats	Rice fields or flooded areas	Tires, bowls, cups, natural basins
	Climate preference	Predominant during dry season [16]	Predominant beginning of the rainy season [17]
**Genome**	Genome size	278 Mb [18]	1380 Mb [19]
	Chromosome length	Shorter	2.3 times longer [20]
	Transposable element composition	16% of the genome [18]	50% of the genome [21]
	Transposable element localisation	Pericentromeric heterochromatin	Euchromatin
	Protein orthology	67% [19]	
	Number of orthologs	2000 [19]	
**Geographic distribution**		Sahara, Northern Europe, Northern Asia	Northern Africa, Australia
		America, South and Sub-Saharan Africa, West and East Asia Western Europe	
**Pathogens transmission**	Parasites	*Plasmodium* spp.	/
		*Wuchereria bancrofti* [22,23]	
	Viruses	ONNV	*Flaviviruses Alphaviruses Phleboviruses Orthobuynyaviruses*
**C. Vectorial system comparison**			
		**ONNV/*Anopheles gambiae***	**CHIKV/*Aedes aegypti***
**Extrinsic incubation period**	Passage of the Midgut barrier	3 days post infection [24]	Before 2 days post infection [25,26,27,28]
	In salivary glands	7 days post infection [24]	2 to 3 days post infection [25,26,27,28]
**Receptors for viral entry**	Putative receptors	230 kDa Cadherin and Rab5 ortholog could be involved in ONNV entry [29]	38 kDa and 60 kDa protein at the membrane brush border of the midgut [30]
		Lectins and prohibitins [29,30,31]	
	Attachment factors	Glycoaminoglycans [31]	
**Antiviral immunity**	RNA interference	siRNA, piRNA and miRNA	
	Toll pathway	Inhibited by virus Repress viral dissemination [24]	Inhibited by virus and no antiviral response [32]
	IMD pathway	Inhibited by virus Protective against ONNV midgut infection (with Rel2-F) [24]	Inhibited by virus and no antiviral response [32]
	JAK/STAT pathway	Inhibited by virus Protective against ONNV midgut infection [24]	Inhibited by virus and no antiviral response [32]
	JNK pathway	No evidence	Antiviral response [33]
	Complement-like pathway	APL1A, APL1C and LRIM4 [24]	TEP20 [33]
	AMP	/	Cecropin-like peptide [34]
	Microbiota	These viruses require enteric microbiome [24,35,36]	

## Data Availability

Data is contained within the article.
